# Anti-spike protein to determine SARS-CoV-2 antibody levels: Is there a specific threshold conferring protection in immunocompromised patients?

**DOI:** 10.1371/journal.pone.0281257

**Published:** 2023-04-28

**Authors:** Philippe Halfon, Sylvie Jordana, Stéphane Blachier, Philippe Cartlamy, Laurent Kbaier, Christina K. Psomas, Patrick Philibert, Gilles Antoniotti, Julie Allemand-Sourrieu, Stanislas Rebaudet, Guilhem Cavaille, Chloé Stavris, Frédérique Retornaz, Laurent Chiche, Guillaume Penaranda

**Affiliations:** 1 Laboratoire Alphabio–Biogroup, Marseille, France; 2 Department of Infectious Diseases and Internal Medicine, Hôpital Européen, Marseille, France; 3 Laboratoires Oriade Noviale–Biogroup, Grenoble, France; 4 Laboratoire Bioestérel–Biogroup, Mouans-Sartoux, France; 5 Nosoconseil, Aix les Bains, France; Elazıg Fethi Sekin City Hospital: Elazig Fethi Sekin Sehir Hastanesi, TURKEY

## Abstract

**Background:**

Identifying a specific threshold level of SARS-CoV-2 antibodies that confers protection in immunocompromised patients has been very challenging. The aim was to assess the threshold of 264 binding antibody units (BAU)/ml using four different SARS-CoV-2 antibody assays (Abbott, Beckman, Roche, and Siemens) and to establish a new optimal threshold of protection for each of the four antibody assays.

**Methods:**

This study was performed on data retrieved from 69 individuals, who received at least one dose of the Pfizer/BioNTech BNT162b2 or Moderna COVID-19 vaccine (Spikevax) at the Alphabio Laboratory in Marseille, France (European Hospital, Alphabio–Biogroup). The results were compared to the percent inhibition calculated using a functional surrogate of a standardized virus neutralization test (Genscript).

**Results:**

Samples from 69 patients were analyzed. For a reference cutoff of 264 BAU/ml, assays showed moderate to good overall concordance with Genscript: 87% concordance for Abbott, 78% for Beckman, 75% for Roche, and 88% for Siemens. Overall concordance increased consistently after applying new thresholds, i.e., 148 BAU/ml (Abbott), 48 (Beckman), 559 (Roche), and 270 (Siemens).

**Conclusion:**

We suggest specific adjusted thresholds (BAU/ml) for the four commercial antibody assays that are used to assess pre-exposure prophylaxis in immunocompromised patients.

## Introduction

As with many viral respiratory infections, knowledge of the immune response to SARS-CoV-2 after a natural infection or vaccination, that could be predictive of the protection conferred, is challenging and not well established [1–4]. To date, few studies have defined correlates of protection against SARS-CoV-2 infection that can be used by regulators and vaccine developers. Protection against COVID-19 is thought to depend on the presence of specific antibodies against the virus, as well as the function of other components of the immune system such as T cells. Furthermore, the immunity in immunocompromised individuals may be less robust than in healthy individuals and may wane more quickly. It is still being studied how does the immune system react in immunocompromised individuals, and how these observations translate into protection. The threshold or cutoff value for immunity in immunocompromised individuals in relation to COVID-19 is currently not well established. Increasing evidence suggests that vaccination regimens for COVID-19, that are applied to the general population, do not adequately protect a significant proportion of immunocompromised patients [5,6]. It’s worth to mention that, as of now, there’s no widely accepted cutoff value for immunity in immunocompromised patients, but some studies have suggested that antibody levels cut off may be associated with protection against COVID-19.

A recent randomized efficacy trial of the ChAdOx1 nCoV-19 (AZD1222) vaccine conducted in more than 8,500 patients in the United Kingdom, analyzed the antibody levels associated with protection against SARS-CoV-2 [7]. They concluded that higher levels of all immune markers were correlated with a reduced risk of symptomatic infection. A vaccine efficacy of 80% was achieved with 264 binding antibody units (BAU)/ml (95% confidence interval [CI]: 108, 806) for anti-spike, and 506 BAU/ml (95% CI: 135, over limit) for anti- receptor-binding domain (RBD) antibodies. Recommendations based on only one study is not prudent. Indeed, the BAU/ml values were performed only on the B.1.1.7 variant in neutralization assays and not on different strains of the virus; hence, there may be no relation between immune markers and disease outcome [7].

Anti-SARS-CoV-2 antibody therapies have proven to be efficient in preventing hospitalization in unvaccinated high-risk patients, when administered early on after polymerase chain reaction (PCR) diagnosis or after contact with infected individuals [8]. Indeed, antibody therapy for pre-exposure prophylaxis (PrEP), may be efficient in preventing hospitalization in immunocompromised patients, regardless of the variant involved.

Based on these studies, a threshold of 264 BAU/ml antibody was used as a recommendation for the use of PrEP in SARS-CoV-2 in France, and extrapolated to immunocompromised patients [9].

Few studies have highlighted the lack of standardization of SARS-CoV-2 serology, despite the use of the international standards set by the World Health Organization (WHO) for SARS-CoV-2 immunoglobulin levels (BAU/ml) [10–13].

The objective of the present study was to establish a new optimal threshold of protection for four different SARS-CoV-2 antibody assays [14].

## Materials and methods

### Study design and participants

This study was performed using sera collected between October 2021 and December 2021 from a real life cohort of 69 individuals attending internal medicine and infectious diseases department of the European Hospital (Marseille). All samples were collected at the Alphabio Laboratory in Marseille, France (European Hospital, Alphabio–Biogroup). Inclusion criteria were data from immunocompromised patients undergoing chemotherapy and/or biotherapy, aged over 18, who received at least one dose of the Pfizer/BioNTech BNT162b2 or Moderna COVID-19 vaccine (Spikevax) from three to six months before sampling collection.

According to French regulations, the study was approved by the French ethics committee (Health Data Hub, approval number: F20211217094518). The ethics committee waived the need for formal written informed consent from patients, as this study was performed on clinical data retrieved from routine tests; thus, no patient was specifically included in this study. As required by French regulations, patients attending clinical laboratories are informed that their biological results can be used for research purposes and that they are free to refuse to allow this (information annotated in the clinical laboratory report). All data were fully anonymized before the analysis. This study complied with the World Medical Association Declaration of Helsinki regarding the ethical conduct of research involving human subjects.

### Laboratory procedures

Four antibody binding assays were used for serological testing according to the instructions of the manufacturer. Two were quantitative: Abbott SARS-CoV-2 IgG II Quant-test (Abbott) (Abbott France, Rungis, France) with 50 arbitrary units (AU)/ml as a threshold for positivity, and Roche Elecsys anti-SARS-CoV-2 S (Roche Diagnostics France, Meylan, France) with 0.8 AU/ml used as a threshold for positivity. Two were semi-quantitative: Beckman Access SARS-CoV-2 IgG II (Beckman Coulter France SAS, Roissy CDG, France) with 30 AU/ml as a threshold for positivity and Siemens Atellica® IM SARS-CoV-2 IgG (Siemens Healthcare SAS, Saint-Denis, France) with 0.8 AU/ml used as a threshold for positivity.

BAU/ml proposed by the WHO, to standardize any assay to the WHO International Standard, were calculated by applying the following conversion factors as suggested by the manufacturers: Abbott, BAU/ml = (1/7) × Antibody Units (AU)/ml, Beckman, BAU/ml = 1 × AU/ml, Roche, BAU/ml = 1.029 × AU/ml, and Siemens, BAU/ml = 21.8 × AU/ml.

The neutralizing capacity was estimated by performing a surrogate virus neutralization test (sVNT) assay (GenScript, Piscataway, NJ, USA) as previously described [10,15,16]. This assay detects antibodies that block the interaction of SARS-CoV-2 with its entry receptor angiotensin-converting enzyme 2. A threshold of 20% was used for positivity.

### Statistical analyses

Quantitative data were reported using median and interquartile range (IQR), and qualitative data were reported using frequency and percentage. The nonparametric Kruskal–Wallis test for multiple comparisons was used to compare all assays. Pairwise comparisons were performed using the nonparametric Wilcoxon test. Agreements between antibody-binding assays and Genscript sVNT were performed using Cohen’s kappa, crude concordance rate, and area under curve (AUC). Optimal cutoffs for distinguishing positivity were calculated using logistic regression on Genscript sVNT binary results (negative/positive), prior to the Youden index maximization approach on receiver operating characteristic curve results. The Youden index indicates the performance (the larger the better) at a given cutoff: Youden = sensitivity + specificity– 1 (the maximum value of the Youden index is 1) [17]. Statistical significance was set at P < 0.05. Calculations were performed using the SAS V9.4 software (SAS Institute Inc., Cary, NC, USA).

## Results

Samples from 69 patients were included in this study. Baseline characteristics are shown in Table 1. The female/male ratio was 67/33, and the median age was 47 years (IQR 34–63). All patients had received at least one dose of either Pfizer/BioNTech BNT162b2 or Moderna COVID-19 vaccine (Spikevax): 60 patients received Pfizer vaccine (87%) and 9 received Moderna vaccine (13%). Vaccination status was complete among 61 patients (88%). Median time between last vaccination and sampling was 5.2 months (3.1–6.4).

**Table 1:** Baseline characteristics.

Baseline Characteristics	
Age (years)–Median (q1-q3)	47 (34–63)
Gender–N (%) *Female* *Male*	46 (67%) 23 (33%)
Vaccine Type–N (%) *Pfizer* *Moderna*	60 (87%) 9 (13%)
Vaccination Status–N (%) *Complete* *Incomplete* *If complete status*, *how many doses *?* *: * 2 doses* *3 doses*	61 (88%) 8 (12%) 40 (66% of 61) 21 (34% of 61)
Time between last vaccination and sampling (months)–Median (q1-q3)	5.2 (3.1–6.4)
Biotherapy–N (%)	
Rituximab	25 (36%)
Cortisone	18 (26%)
Plaquenil (Hydroxychloroquine)	14 (20%)
Immunosuppressive	43 (62%)

The median values observed for the antibody binding assays were 143 BAU/ml (IQR 39–748) for Abbott, 55 BAU/ml (IQR 19–217) for Beckman, 636 BAU/ml (IQR 98–2369) for Roche, and 161 BAU/ml (IQR 32–574) for Siemens, which demonstrated the variations between the assays (overall P < 0.0001). Beckman assay showed lower values as compared to all other assays (P< 0.008 for all paired comparisons); and lower values was observed for Siemens assay compared with Roche assay (P = 0.0033).

**Fig 1:**
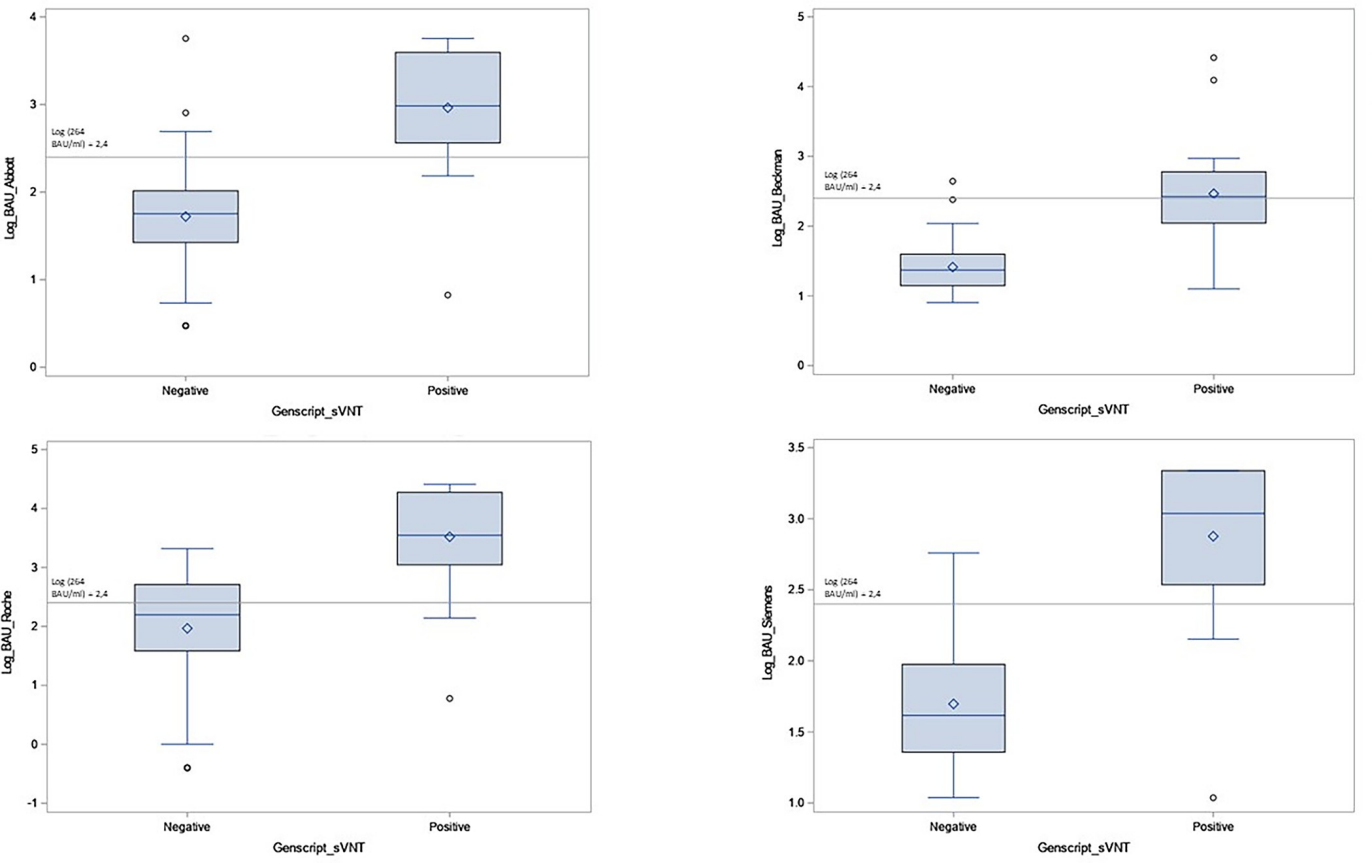
Boxplots for each antibody binding assay according to Genscript sVNT positive and negative results. Solid reference line represents 264 binding antibody units (BAU)/ml cutoff (2.4 Log). The Wilcoxon test for pairwise comparisons yielded P < 0.0001 for all comparisons.

**Fig 2:**
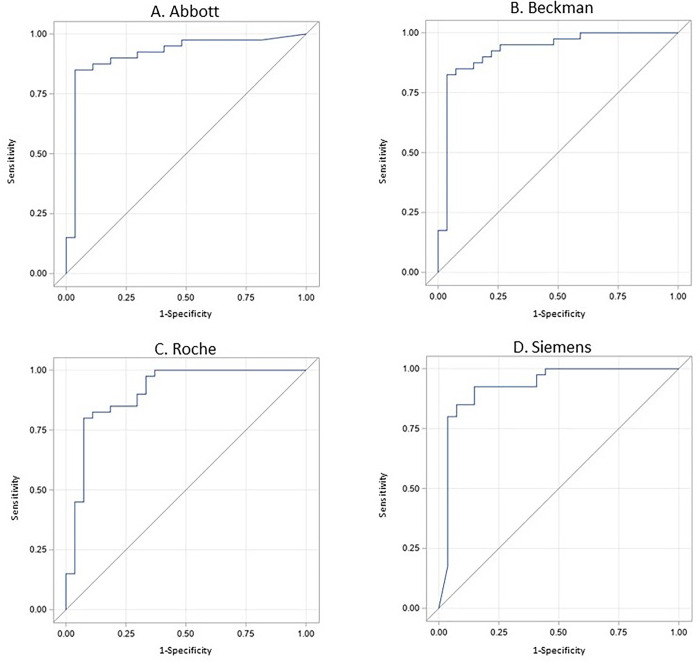
ROC curves for each antibody binding assay according to Genscript sVNT.

Comparisons were performed between Genscript sVNT positive and negative samples according to antibody binding assays, all of which were significant (P < 0.0001) (Fig 1). Agreement between the antibody binding assays and the Genscript sVNT assay is shown in Table 2. When considering a reference cutoff of 264 BAU/ml, the assays showed moderate to good agreement with Genscript sVNT, with strong variations of the kappa index from 0.52 for Beckman and Roche to 0.76 for Siemens (kappa = 0.72 for Abbott). The overall concordance between antibody binding assays and the Genscript sVNT varied from 75% for Roche to 88% for Siemens (87% for Abbott and 78% for Beckman). All assays showed a high AUC for prediction of positive and negative results of Genscript sVNT (AUC > 0.90 for all) (Fig 2).

**Table 2:** Agreement between antibody binding assays and Genscript sVNT positive and negative results according to the reference cutoff (264 BAU/ml).

Assay	Genscript sVNT (n)		Kappa Index [95%CI]	AUC [95%CI]
	Negative	Positive		
**Abbott** *	36	5	0.72 [0.55,0.89]	0.91 [0.83,0.99]
	4	22		
**Beckman**	39	14	0.52 [0.33,0.72]	0.93 [0.86,1.00]
	1	15		
**Roche**	25	2	0.52 [0.34,0.71]	0.91 [0.83,0.99]
	15	27		
**Siemens**	36	4	0.76 [0.61,0.92]	0.93 [0.85,1.00]
	4	25		

AUC, Area under curve; sVNT surrogate virus neutralization test

*n = 67, two results could not be assessed by the Abbott antibody binding assay.

The optimal cutoff was analyzed for each antibody binding assay (Table 3). Using the Youden index maximization approach, optimal cutoffs were consistently lower than the reference cutoff of 264 BAU/ml for the Abbott and Beckman assays (148 and 48 BAU/ml, respectively). In contrast, the optimal cutoff was higher for the Roche assay (559 BAU/ml). For the Siemens assay, the optimal cutoff was within the same range as the reference cutoff (270 BAU/ml). When considering specific optimal cutoffs, agreement between each antibody binding assay and Genscript sVNT increased consistently from 0.03 units for the Siemens assay to 0.25 units for the Beckman assay (kappa = 0.79 and 0.77, respectively). Kappa increased to 0.76 for the Abbott assay (0.04 units increase) and to 0.71 for the Roche assay (0.19-unit increase). Overall, all assays showed good agreement with the Genscript sVNT. The overall concordance between the antibody binding assays and the Genscript sVNT also increased consistently i.e., 11% increase for Roche (86% concordance), 10% increase for Beckman (88% concordance), 2% increase for Siemens (90% concordance), and 1% increase for the Abbott assay (88% concordance). A subgroup analysis was performed according to vaccination status (complete or incomplete). Previous specific optimal cutoffs fitted perfectly to patients with incomplete vaccination: a perfect agreement was observed between Genscript sVNT and each antibody binding assays among these patients (results not shown). No significant difference among agreements was observed.

**Table 3:** Agreement between antibody binding assays and Genscript sVNT positive and negative results according to optimal cutoff values determined for each assay.

Assay	Optimal Cutoff* (BAU/ml)	Genscript sVNT (n)		Kappa Index [95%CI]
		Negative	Positive	
**Abbott** **	<148	33	1	0.76 [0.61,0.91]
	≥148	7	26	
**Beckman**	<48	33	1	0.77 [0.62,0.92]
	≥48	7	28	
**Roche**	<559	32	2	0.71 [0.55,0.87]
	≥559	8	27	
**Siemens**	<270	37	4	0.79 [0.54,0.94]
	≥270	3	25	

BAU, binding antibody units; AUC, Area under curve; sVNT surrogate virus neutralization test

* Optimal cutoff determined using Youden index maximization

**n = 67, two results could not be assessed by the Abbott antibody binding assay.

## Discussion

The use of antibody therapy for PrEP, which is the use of medications to prevent infection before exposure to a virus, is currently being studied for its potential efficacy in immunocompromised individuals with COVID-19. There are currently a few monoclonal antibody cocktails (such as bamlanivimab, casirivimab, and imdevimab together) that have been authorized by the US FDA for emergency use for the treatment of COVID-19 in certain population and similar medications have been authorized in other countries. These medications are primarily indicated for individuals who are at high risk of severe illness or death from COVID-19, including those who are immunocompromised. Clinical studies are ongoing to evaluate the effectiveness and safety of these medications in immunocompromised individuals and using them as PrEP. So there is not enough data available to comment on the uptake of this therapy yet and raises the question in cases of previous infection or vaccination, the need to assess the SARS-CoV-2 antibody level for therapy decision making [18–20].

The use of a specific threshold for decision-making regarding PreP in immunocompromised patients must be taken with cautions due to limitations, mainly based on the nature and type of assay used to measure the antibody. As previously observed by Perkmann et al. although all assays showed good agreement with the Genscript sVNT, they were not interchangeable, even when converted to BAU/ml [10]. The differences in the commercial assays used in this study are related to the components of the tests (the spike antigen epitopes used, the different isolates of the SARS-CoV-2, and the quantification of either total antibodies or only IgG) [21–23]. Indeed, cutoff values established using commercially available SARS CoV-2 diagnostic antibodies cannot represent a “gold standard” threshold value related to a level of neutralizing activity. Therefore SARS-CoV-2 serology may be standardized.

For SARS-CoV-2, tests to neutralize live viruses are performed only in specialized laboratories and are not standardized, making it difficult to compare and justify the use of a well-characterized sVNT as a functional reference [24,25].Additionally, neutralizing antibodies were not investigated, which could have helped in determining whether the anti-RBD or the anti-spike assays had the strongest correlation with virus neutralization. However, harmonization of neutralizing antibody titers is necessary to determine a common threshold using which vaccine protection can be predicted. This would allow for identification of the corresponding thresholds, using high-throughput binding antibody assays.

There were few limitations in this study. One of these was the low number of samples that were subjected to antibody quantification and the absence of an independent international standard (WHO in IU/ml). Another limitation was the lack of an external cohort to validate the suggested thresholds. There is also a limitation regarding the two semi-quantitative antibody binding assays as a saturation limit could be reached because of their limited measurement range. Another important limitation is that samples were collected at any time after the last vaccine dose (median 5.2 months (3.1–6.4)); Swadzba et al. showed time-dependent changes in the comparability of different antibody tests with samples collected at different time points [26].

In conclusion, whether it is generally believed that a certain level of antibodies is necessary to confer protection against the virus, but the exact level required is not yet clear. Different studies have used different methods to measure antibody levels, making it difficult to compare results and establish a universal cutoff value conferring protection in immunocompromised patients. Therefore, we suggest specific BAU/ml adjusted thresholds for the four commercial antibody assays (Abbott, Beckman, Roche, and Siemens), which can be used to guide the use of PreP in immunocompromised patients.

## Supporting information

S1 Data (CSV)
